# Pulmonary immune profiling of SIDS: impaired immune maturation and age-related cytokine imbalance

**DOI:** 10.1038/s41390-022-02203-8

**Published:** 2022-08-19

**Authors:** Dong Qu, Theresa A. Engelmann, Vanessa Preuss, Lars Hagemeier, Lena Radomsky, Kerstin Beushausen, Jana Keil, Benedikt Vennemann, Christine S. Falk, Michael Klintschar

**Affiliations:** 1 Institute of Legal Medicine, Hannover Medical School, Hannover, Germany; 2 Institute of Transplant Immunology, Hannover Medical School, Hannover, Germany; 3 German Center for Infection Research, DZIF, TTU-IICH, Hannover-Braunschweig site, Hannover, Germany

## Abstract

**Background:**

For sudden infant death syndrome (SIDS), an impaired immunocompetence has been discussed for a long time. Cytokines and chemokines are soluble immune mediators (SIM) whose balance is essential for the immune status. We hypothesized that an imbalanced immune response might contribute to the etiology of SIDS.

**Methods:**

We investigated 27 cytokines, chemokines, and growth factors in protein lysates of lungs derived from 29 SIDS cases and 15 control children deceased for other reasons.

**Results:**

Except for the CCL5, no significant differences were detected in the lungs between SIDS cases with and without mild upper respiratory tract infections. In contrast, IL-1RA, IL-7, IL-13, and G-CSF were decreased in the merged SIDS cases compared to control cases without evidence of infection. Plotting SIM concentrations against infant age resulted in increasing concentrations in control but not in SIDS lungs, indicating a disturbed immune maturation. Moreover, an age-dependent shift towards a Th2-related pattern was observed in SIDS.

**Conclusions:**

Our findings suggest that an impaired maturation of the immune system, an insufficient response to respiratory pathogens, and an immune response modulated by Th1/Th2 imbalance might play a possible role in triggering SIDS. These findings might in part be explained by chronic stress.

**Impact:**

- Maturation of the cytokine and chemokine network may be impaired in SIDS.
- An imbalance between Th1- and Th2-related cytokines, which may reflect a state of chronic stress causing a more Th2 shift.
- An impaired immune maturation, an insufficient response to respiratory pathogens, and an immune response modulated by Th1/Th2 imbalance might play a possible role in SIDS.

## Introduction

Sudden infant death syndrome (SIDS)^1^ is the leading cause of death between 1 month and 1 year of age among infants in the developed countries. The triple risk model suggests that a combination of a critical development stage, a vulnerable infant, and exogenous factors is linked to SIDS.^2^ Nevertheless, the specific etiologic mechanism remains unclear.

Among the numerous etiologies discussed in the context of SIDS, various predisposing factors associated with inflammatory reactions have been recognized for a long time: mild infection in the respiratory tract prior to death is frequently observed and might trigger a cascade leading to SIDS via an imbalanced immune response.^3–7^ Hence, numerous, in part contradictory, studies have been performed by measuring inflammatory cells, immunoglobulins, or cytokines in upper respiratory tracts and lungs^8–15^ or typing gene polymorphisms.^16–20^ These controversial results are major hurdles for hypotheses explaining whether an altered immunological homeostasis may occur in the respiratory system and how it might affect the infant’s physiology and organ function. It has been speculated that dysregulated cytokines or other soluble immune mediators (SIMs) may lead to SIDS via respiratory control, hyperthermia, vascular shock, and cardiac arrhythmia.^21^ To our knowledge, only two previous studies have investigated the cytokine expression in the lungs in SIDS. One study tested only one cytokine (interleukin (IL)-2) and failed to demonstrate this protein in post-mortal lung bronchioalveolar lavage fluid.^10^ The other study investigated several cytokines at the mRNA level using reverse transcriptase–polymerase chain reaction and the protein level via immunocytochemistry but is available as abstract only.^13^ Therefore, the magnitude of alterations of SIMs in the lungs of SIDS victims remains largely unknown.

Immune regulation involves several mechanisms, among which the balance between T helper 1 (Th1) and T helper 2 (Th2) activity is one of the most widely accepted.^22,23^ The Th1 response is related to the pro-inflammatory response, which is essential to mount a protective response against infections, while the Th2 response is more associated with a negative feedback of inflammatory responses, allergic responses, and humoral immunity.^24^ Measurement of the cytokine/chemokine network allows to distinguish between Th1 and Th2 predominance.

We thus hypothesized that a dysregulated immune response, possibly in combination with a respiratory infection, may be involved in the etiology of SIDS. Therefore, our study aimed to investigate the cytokine/chemokine network by profiling 27 SIMs in lung tissue of SIDS vs. deceased control children to further explore potential mechanisms of pulmonary inflammation in SIDS.

## Materials and methods

### Study participants

The subjects investigated were 44 control and SIDS cases autopsied at the Institute of Legal Medicine, MHH, between 2010 and 2020. Approval was given by the local ethics committee (No. 1211–2011). All the cases were categorized into four groups based on the cause of death. Control^−^ group: controls without signs of infection; control^+^: controls with signs of infection; SIDS^−^ group: SIDS cases without evidence of infection; SIDS^+^ group: SIDS cases with evidence of mild upper respiratory tract infection. For none of the SIDS cases, specific causes of death were determined after thorough postmortem examinations and death scene investigations. Detailed information (cause of death, age, sex, infection, circumstances around the death, and related risk factors) of control and SIDS cases is given in Tables 1 and 2. Lung tissues were sampled during the autopsy and were stored at −80 °C for long-term storage.

**Table 1:** Control cases with detailed information.

Coded case no.	Group	Gender	Age (weeks)	Postmortem interval (days)	Storage period (years)	Cause of death	Infection duration in history (days)
C1-2	Control^−^	Female	1	2	9	Heart defect	—
C2-2	Control^−^	Male	17	2	9	Trauma	—
C4-2	Control^−^	Male	1	3	9	Heart defect	—
C6-2	Control^−^	Male	21	2	9	Heart defect	—
C9-2	Control^−^	Female	13	1	8	Trauma	—
C13-2	Control^−^	Female	25	2	5	Metabolic disorder (LCHAD defect)	—
C-S16-2	Control^−^	Female	10	1	8	Trauma and cardiomyopathy	—
C14-2	Control^−^	Male	9	2	2	Trauma	—
C20-2	Control^−^	Male	65	3	1	Trauma	—
C21-2	Control^−^	Male	0	2	1	Premature placental ablation	—
C8-2	Control^+^	Male	15	1	9	Trauma and aspiration pneumonia	2
C10-2	Control^+^	Male	23	3	8	Aspiration pneumonia	2
C3-2	Control^+^	Female	97	2	9	Sepsis	2
C7-2	Control^+^	Male	8	1	9	Sepsis	3
C12-2	Control^+^	Female	63	4	8	Sepsis	4

**Table 2:** SIDS cases with detailed information.

Coded case no.	Group	Gender	Age (weeks)	Postmortem interval (days)	Storage period (years)	Autopsy findings	Clinical infection history	Infection duration in history	Sleeping position	Co-sleeping	Maternal smoking	Other risk factors
S1-2	SIDS^−^	Male	12	2	10	—	—	—	Back	No	Unknown	Twin/preterm (33 weeks)
S2-2	SIDS^−^	Female	22	2	10	—	—	—	Prone	No	Yes	—
S3-2	SIDS^−^	Male	13	1	9	—	—	—	Prone	No	Unknown	—
S7-2	SIDS^−^	Female	3	2	9	—	—	—	Prone	No	Unknown	—
S9-2	SIDS^−^	Male	14	4	9	—	—	—	Prone	Yes	Unknown	—
S10-2	SIDS^−^	Female	36	1	9	—	—	—	Prone	No	Unknown	Preterm
S11-2	SIDS^−^	Male	7	2	9	—	—	—	Back	No	Unknown	Preterm
S13-2	SIDS^−^	Female	19	2	8	—	—	—	Prone	No	Unknown	Preterm (26 weeks)
S15-2	SIDS^−^	Male	5	1	8	—	—	—	Back	Yes	Unknown	—
S18-2	SIDS^−^	Male	9	2	8	—	—	—	Unknown	No	Unknown	—
S19-2	SIDS^−^	Male	33	2	8	—	—	—	Prone	No	Unknown	—
S22-2	SIDS^−^	Male	18	2	7	—	—	—	Prone	No	Unknown	—
S23-2	SIDS^−^	Male	12	2	7	—	—	—	Prone	No	Unknown	—
S25-2	SIDS^−^	Female	11	1	6	—	—	—	Prone	No	Unknown	—
S26-2	SIDS^−^	Male	10	2	6	—	—	—	Back	No	Yes	—
S27-2	SIDS^−^	Male	30	2	6	—	—	—	Prone	No	Unknown	—
S28-2	SIDS^−^	Male	23	4	6	—	—	—	Back	Yes	Unknown	—
S29-2	SIDS^−^	Male	23	1	6	—	—	—	Prone	No	Unknown	—
S30-2	SIDS^−^	Female	8	2	6	—	—	—	Prone	No	Unknown	—
S32-2	SIDS^−^	Male	17	2	3	—	—	—	Side	No	Unknown	—
S4-2	SIDS^+^	Male	17	2	9	Tracheobronchitis	—	—	Prone	No	Unknown	Preterm (36 weeks)
S5-2	SIDS^+^	Female	28	2	9	Otitis media	Otitis media	2 weeks	Prone	No	Unknown	—
S8-2	SIDS^+^	Female	14	2	9	—	Respiratory infection	4 weeks	Back	No	Unknown	—
S12-2	SIDS^+^	Male	6	3	8	Tracheobronchitis	Fever	2 days	Prone	No	Unknown	—
S14-2	SIDS^+^	Male	19	2	8	Tracheobronchitis	Respiratory infection	10 days	Prone	Yes	Yes	—
S17-2	SIDS^+^	Male	19	2	8	—	Respiratory infection	2 weeks	Back	No	Unknown	—
S20-2	SIDS^+^	Female	13	1	8	—	Rota virus infection	3 weeks	Back	No	Unknown	—
S21-2	SIDS^+^	Male	16	3	7	Tracheobronchitis	Respiratory infection	1 week	Prone	Yes	Unknown	—
S24-2	SIDS^+^	Male	29	1	6	Tonsillitis	—	—	Prone	No	Unknown	—

### Measurement of SIMs using multiplex arrays

Protein was extracted from 3 mm × 3 mm specimens of the lung tissue using the Bio-Rad cell lysis Kit (#171304011, BioRad, Hercules, CA). Protein concentration was determined by Pierce^TM^ BCA Protein Assay Kit (#23225, Thermo Scientific, Waltham, MA), and was adjusted to 1000 μg/mL using Sample Diluent (BioRad, Hercules, CA). The concentrations of SIMs were measured in lung protein lysates containing 50 µg protein using Bio-Plex Pro^TM^ Human Cytokine 27-Plex Assay (#500KCAF0Y, BioRad, Hercules, CA) in accordance with the manufacturer’s instruction. Standard curves and concentrations were calculated with Bio-Plex-Manager 6.2 software (BioRad, Hercules, CA). Concentrations below the lower limit of detection (LLOD) were assigned the value of the LLOD, concentrations beyond the upper limit of detection (ULOD) assigned the value of the ULOD.

### Heatmap cluster analysis and principal component analyses (PCAs)

The heatmap was generated using R (version 4.0.4). SIM concentrations were log-transformed (mean = 0, variable 1) and scaled in the level of each SIM. The SIDS and control cases were assigned into 4 subgroups by columns, and the cytokine markers were classified into 4 functional subgroups by rows. The red color represents high-expressed and the blue color low-expressed analytes. Moreover, to assess the differences among the 4 different groups in this study, the PCA was utilized to cluster the samples based on their similarity using R (version 4.0.4).

### Statistical analysis

The distribution of variables was analyzed with the Shapiro–Wilk (S-W) normality test. Quantitative data of the original cytokine/chemokine concentrations were expressed in the form of medians and ranges due to the non-normal distribution data. A Kruskal–Wallis one-way ANOVA with Dunn’s correction was used for multiple comparisons, and a Mann–Whitney *U*-test was used to compare two groups of continuous variables. A Chi-square (*χ*^2^) test was used to calculate statistical significance for sex proportion in four subgroup groups. A Spearman’s rank correlation analysis (for non-normal distribution data) was used to test for correlation between age and cytokine concentrations. To get a general overview of the age-related changes in the cytokine/chemokine milieu in human lungs in young infants, average values of mean-centered scaled cytokine concentrations were utilized. To that end, we calculated the mean value for all samples for each cytokine and expressed the individual values in percentage of the mean. Then, the sum for all cytokines was calculated for each sample. A *p* value < 0.05 (two-tailed) was accepted as indicating statistical significance. All statistical analyses were performed using the SPSS 24.0 software (SPSS Inc. Chicago) or R (version 4.0.4).

## Results

There were no significant differences in age (Median: 11 vs. 23 vs. 14 vs. 17 weeks, *p* > 0.05), the composition of sex (Male proportion: 60% vs. 60% vs. 70% vs. 67%, *p* > 0.05), the postmortem interval (Median: 2 vs. 2 vs. 2 vs. 2 days, *p* > 0.05), and the sample storage period (Median: 8 vs. 8 vs. 8 vs. 8 years, *p* > 0.05) between the Control^−^, Control^+^, SIDS^−^, and SIDS^+^ groups (Table 3).

**Table 3:** Summary of participant demographics.

Characteristics	Control^−^	Control^+^	SIDS^−^	SIDS^+^	*p* value
Number of cases	10	5	20	9	-
Age in weeks, median (range)	11 (0–65)	23 (8–97)	14 (3–36)	17 (6–29)	*p* > 0.05
Male gender, %	60%	60%	70%	67%	*p* > 0.05
Postmortem interval in days, median (range)	2 (1–3)	2 (1–4)	2 (1–4)	2 (1–3)	*p* > 0.05
Sample storage period in years, median (range)	8 (1–9)	8 (8–9)	8 (3–10)	8 (6–9)	*p* > 0.05

The SIMs included in this study are given in Table 4 and were classified into 4 subgroups according to the function in inflammatory responses. The ratio of Th1/Th2 was calculated by involved cytokines (Th1 cytokines: IL-1β, IL-2, IL-8/CXCL8, IL-12, TNF-α, and IFN-γ; Th2 cytokines: IL-4, IL-5, IL-10, and IL-13). The raw data for the concentrations of SIMs are listed in Supplementary Table S1.

**Table 4:** 27 SIMs included in the study.

Pro-inflammatory cytokines	Pro-inflammatory chemokines	Anti-inflammatory mediators	Growth factors
IL-1β	CCL1 (MCP-1)	IL-1RA	FGF basic
IL-2	CCL3 (MIP-1α)	IL-4	G-CSF
IL-6	CCL4 (MIP-1β)	IL-5	GM-CSF
IL-7	CCL5 (RANTES)	IL-9	PDGF-bb
IL-12 (p70)	CCL11 (Eotaxin)	IL-10	VEGF
IL-15	CXCL10 (IP-10)	IL-13	
IL-17	CXCL8 (IL-8)		
IFN-γ			
TNF-α			

**Fig. 1:**
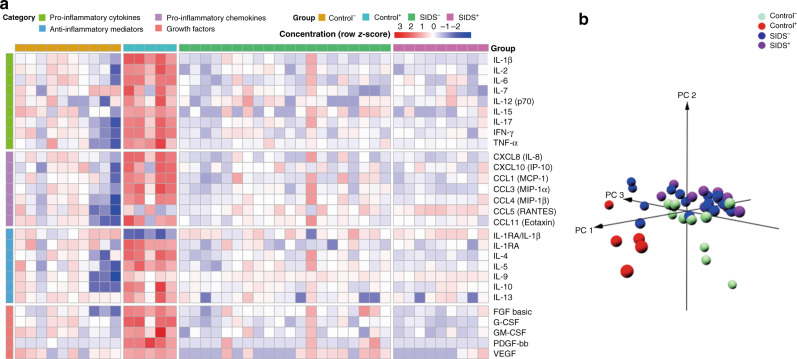
Computational visualization of cytokine expression in four groups of Control^−^, Control^+^, SIDS^−^, and SIDS^+^. **a** Heatmap based on cytokine, chemokine, and growth factor concentrations in the lungs. **b** A principal component analysis (PCA) was displayed in a three-dimension (3D) plot of the four groups.

To display the differences of SIMs in lung tissue, heatmap and PCAs were utilized (Fig. 1). The PCA plot clearly indicated that Control^+^ samples are different from the other groups and separate into a unique cluster. While Control^−^ samples appeared to differ from both SIDS groups, SIDS^−^ and SIDS^+^ samples seemed not to differ from each other despite the documented infections in the SIDS^+^ group. Detailed statistical analyses revealed no significant differences for these proteins except for the pro-inflammatory chemokine CCL5, (Table 5) that was higher in the SIDS^+^ group (*p* = 0.020). Since otherwise no significant differences and, upon histologic examination of the lungs (data not shown), no signs of infection were found, both groups were merged into one new group (“SIDS”) for further analysis.

**Table 5:** Statistical differences in the SIM level of SIDS^−^ and SIDS^+^ cases.

SIMs	Concentration (pg/mL)				Trends for SIDS^+^, compared to SIDS^−^	*p* value
	SIDS^−^ (*n* = 20)		SIDS^+^ (*n* = 9)			
	Median	Range (min.–max.)	Median	Range (min.–max.)		
IL-1RA/IL-1β	649.13	14.13–3307.89	865.37	421.86–1166.50		0.183
IL-1β	2.50	1.13–102.93	2.23	1.50–4.30		0.799
IL-1RA	1637.73	480.40–4714.64	2302.13	660.04–2775.29		0.417
IL-2	1.35	0.21–11.81	0.90	0.21–1.57		0.153
IL-4	0.81	0.37–4.62	0.58	0.45–0.92		0.085
IL-5	113.05	35.72–370.81	89.33	83.28–136.2		0.216
IL-6	11.75	1.51–966.13	51.23	3.41–168.71		0.501
IL-7	4.10	1.36–10.86	4.54	1.36–10.04		0.799
CXCL8 (IL-8)	47.72	11.94–4710.41	59.47	17.96–188.93		0.945
IL-9	108.33	81.34–128.01	115.87	97.81–123.42		0.140
IL-10	1.74	1.12–2.69	1.74	1.12–2.37		0.835
IL-12 (p70)	1.96	0.48–6.84	1.48	0.74–4.23		0.908
IL-13	0.38	0.03–1.24	0.48	0.03–0.87		0.729
IL-15	9.30	1.38–73.23	2.75	1.38–35.92		0.340
IL-17	4.30	2.74–23.89	3.85	3.41–8.33		0.729
CCL11 (Eotaxin)	9.79	4.05–96.86	9.94	4.05–14.76		0.835
FGF basic	17.49	3.28–104.41	12.53	7.53–29.98		0.069
G-CSF	126.80	22.47–1463.04	61.24	31.50–251.60		0.183
GM-CSF	1.35	0.21–5.53	0.74	0.38–2.06		0.317
IFN-γ	18.03	7.79–35.65	20.86	6.42–27.03		0.871
CXCL10 (IP-10)	325.33	57.77–2352.20	325.33	78.28–1716.34		0.945
CCL1 (MCP-1)	120.07	35.09–1333.63	131.14	61.8–385.91		0.945
CCL3 (MIP-1α)	21.36	5.75–276.06	11.20	8.65–31.08		0.095
PDGF-bb	1.20	0.91–34.26	1.20	1.20–1.20		0.532
CCL4 (MIP-1β)	149.01	107.57–599.01	146.82	116.63–255.55		0.627
CCL5 (RANTES)	2269.15	981.32–5804.05	3809.19	2418.74–5156.29	↑	**0.020**
TNF-α	25.88	19.46–98.41	28.88	23.24–38.21		0.660
VEGF	17.33	1.80–145.79	1.80	1.80–81.34		0.274

Note: *p* value <0.05 is marked in bold. The Mann–Whitney *U*-test was used to compare two groups of continuous variables.

*SIDS*^**−**^ SIDS cases without evidence of infection, *SIDS*^*+*^ SIDS cases with evidence of mild infection in the anamnesis or autopsy.

**Fig. 2:**
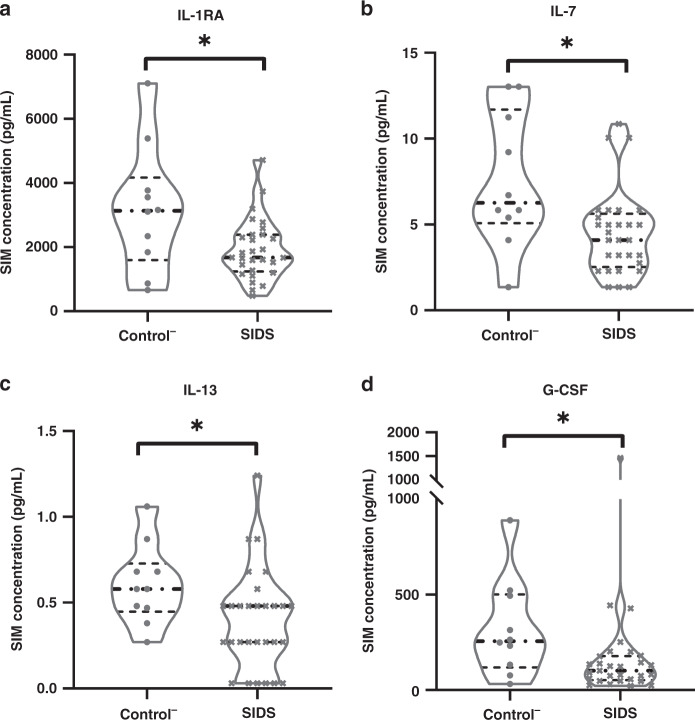
Comparisons of SIM concentrations between the Control^−^ and SIDS groups displayed by violin plots. The black dash line represents the median of SIM concentrations in each group. The dash line in gray represents the first or the third quartile. *p < 0.05.

Differences in the concentrations of these SIMs among the Control^−^, Control^+^, and pooled SIDS groups are shown in Table 6. As expected, nearly all SIMs had elevated concentrations in the Control^+^ group, a pattern dominated by a typical pathogen-driven response via IL-1β, IL-1RA, IL-6, CXCL8 (IL-8), G-CSF, CXCL10 (IP-10), and CCL2 (MCP-1). On the other hand, the concentrations of four SIMs (IL-1RA, IL-7, IL-13, and G-CSF) were significantly lower in the SIDS group, compared to the Control^−^ group (Fig. 2). Besides, most of the other SIMs displayed lower concentrations in SIDS, arguing for a down-regulation, although no statistical significance was reached.

**Table 6:** Statistical differences in the SIM level of control and SIDS cases.

SIMs	Concentration (pg/mL)						Trends and *p* value					
	Control^−^ (*n* = 10)		Control^+^ (*n* = 5)		SIDS (*n* = 29)		Trends for C^+^, compared to C^−^	C^+^ vs. C^−^	Trends for C^+^, compared to S	C^+^ vs.S	Trends for S, compared to C^−^	S vs.C^−^
	Median	Range (min.–max.)	Median	Range (min.–max.)	Median	Range (min.–max.)						
IL-1RA/IL-1β	828.79	145.47–2202.90	7.67	3.43–37.04	685.03	14.13–3307.89	↓	**0.002**	**↓**	**0.001**		0.629
IL-1β	3.79	0.30–19.19	2263.90	199.79–2707.37	2.23	1.13–102.93	↑	**0.002**	↑	**0.000**		0.107
IL-1RA	3136.08	660.87–7109.42	7767.11	7401.40–20,778.91	1674.17	480.4–4714.64	↑	**0.002**	↑	**0.000**	**↓**	**0.046**
IL-2	1.90	0.02–6.11	32.78	8.66–188.35	1.12	0.21–11.81	↑	**0.002**	↑	**0.000**		0.152
IL-4	1.00	0.08–1.99	8.73	1.71–13.07	0.77	0.37–4.62	↑	**0.003**	↑	**0.000**		0.325
IL-5	97.17	9.07–365.73	453.47	309.29–1038.13	104.24	35.72–370.81	↑	**0.005**	↑	**0.001**		0.384
IL-6	35.52	0.13–758.09	20,755.38	536.46–21,612.57	13.54	1.51–966.13	↑	**0.003**	↑	**0.001**		0.210
IL-7	6.27	1.36–13.03	15.71	2.30–62.12	4.10	1.36–10.86	↑	**0.050**	↑	**0.018**	**↓**	**0.011**
CXCL8 (IL-8)	107.73	7.85–763.29	44,278.14	829.43–71,688.14	59.47	11.94–4710.41	↑	**0.002**	↑	**0.001**		0.062
IL-9	97.82	2.17–146.37	131.94	94.20–183.82	109.64	81.34–128.01	↑	**0.049**		0.052		0.088
IL-10	1.74	0.31–3.02	7.07	1.74–23.52	1.74	1.12–2.69	↑	**0.014**	↑	**0.004**		0.782
IL-12 (p70)	1.36	0.18–5.11	4.23	2.28–28.44	1.96	0.48–6.84	↑	**0.017**	↑	**0.003**		0.697
IL-13	0.58	0.27–1.06	1.77	0.48–13.17	0.48	0.03–1.24	↑	**0.023**	↑	**0.002**	**↓**	**0.038**
IL-15	16.49	0.56–88.29	171.27	79.35–295.94	9.30	1.38–73.23	↑	**0.003**	↑	**0.000**		0.279
IL-17	4.52	0.14–10.59	50.76	10.59–95.30	3.85	2.74–23.89	↑	**0.003**	↑	**0.000**		0.884
CCL11 (Eotaxin)	10.72	0.26–25.52	91.24	2.24–529.01	9.94	4.05–96.86	↑	**0.027**	↑	**0.044**		0.974
FGF basic	12.38	1.65–32.12	131.69	78.47–170.62	15.45	3.28–104.41	↑	**0.002**	↑	**0.001**		0.161
G-CSF	256.71	32.57–889.74	31,783.75	287.78–57,468.54	102.40	22.47–1463.04	↑	**0.010**	↑	**0.001**	**↓**	**0.017**
GM-CSF	1.54	0.42–4.98	12.05	3.03–408.78	1.10	0.21–5.53	↑	**0.007**	↑	**0.001**		0.238
IFN-γ	27.03	1.81–54.05	190.31	56–292.09	18.97	6.42–35.65	↑	**0.002**	↑	**0.000**		0.711
CXCL10 (IP-10)	329.55	2.03–3490.47	57,477.58	46–75,793.19	325.33	57.77–2352.2	↑	**0.037**	↑	**0.034**		0.847
CCL1 (MCP-1)	240.48	9.03–510.76	3191.33	715.92–5635.24	122.99	35.09–1333.63	↑	**0.002**	↑	**0.001**		0.351
CCL3 (MIP-1α)	22.52	2.38–81.56	844.03	43.36–2481.59	17.64	5.75–276.06	↑	**0.005**	↑	**0.001**		0.607
PDGF-bb	1.20	1.20–2.91	80.36	27.91–575.39	1.20	0.91–34.26	↑	**0.002**	↑	**0.000**		0.150
CCL4 (MIP-1β)	144.49	6.25–252.97	764.27	149.07–916.27	146.82	107.57–599.01	↑	**0.014**	↑	**0.003**		0.607
CCL5 (RANTES)	1411.45	17.50–6907.93	1042.41	501.18–9570.51	2685.73	981.32–5804.05		0.624		0.296		0.094
TNF-α	24.94	0.38–316.3	560.70	141.18–5662.15	26.25	19.46–98.41	↑	**0.005**	↑	**0.000**		0.376
VEGF	25.65	1.80–137.48	240.69	157.94–334.38	1.80	1.80–145.79	↑	**0.002**	↑	**0.000**		0.243

Note: *p* value <0.05 is marked in bold.

*Control*^*−*^ *(C*^*−*^*)* controls without signs of infection, *Control*^*+*^ *(C*^*+*^*)* controls with signs of infection, *SIDS (S)* sudden infant death syndrome.

**Fig. 3:**
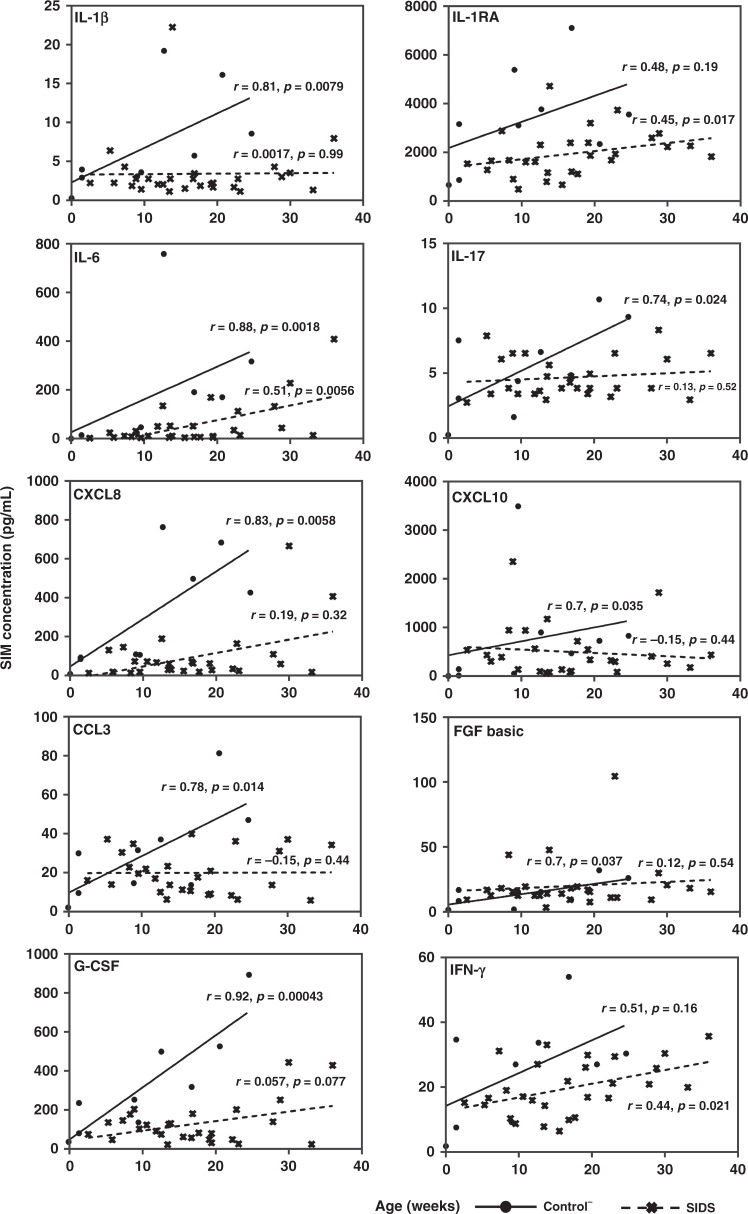
Correlations between age and selected SIMs in the Control^−^ and SIDS groups. *R* in the plot represents the correlation coefficient.

**Fig. 4:**
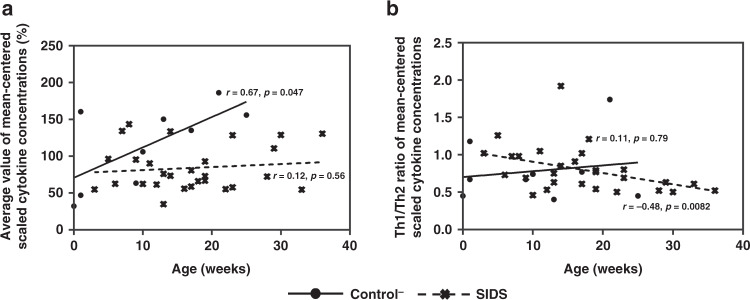
Correlations between age and the average value of all cytokine levels (mean-centered scale) in the Control^−^ and SIDS groups. **a** The correlation between age and the average value of mean-centered scaled cytokine concentrations. **b** The correlation between age and the Th1/Th2 ratio of mean-centered scaled cytokine concentrations.

Selected ratios of Th1- and Th2-associated cytokines with significant discrimination capacity between the Control^−^ and SIDS groups are displayed in Table 7. Eight decreased Th1/Th2 ratios, indicating a Th2 shift, and two increased ratios, arguing for a Th1 shift, were identified in SIDS compared to Control^−^. Based on these results, a pronounced Th2 shift was detectable in SIDS. Moreover, associations between SIM levels and age were examined. For this analysis, only the controls^−^ with an age of less than 52 weeks were used. (Fig. 3). Seven SIMs (IL-1β, IL-17, G-CSF, FGF basic, CCL3, CXCL8, and CXCL10) were strongly positively correlated to age in Control^−^, but not in SIDS. Only IFN-γ and IL-1RA were positively correlated with age in the SIDS group, but not in the Control^−^ group, although older individuals also seemed to have higher levels of these cytokines. IL-6 was positively correlated with age in both the Control^−^ and SIDS groups, although the correlation coefficient in the Control^−^ group was twice that of the SIDS group. When plotting the normalized means for all SIMs against age, they increased in the Control^−^ group (*r* = 0.67, *p* = 0.047), but not in the SIDS group (*r* = 0.12, *p* = 0.56) (Fig. 4a). The same principle was used for the Th1/Th2 ratios of mean-centered scaled cytokine concentrations that were negatively correlated with increasing age in the SIDS group (*r* = −0.48, *p* = 0.0082), but not in the Control^−^ group (*r* = 0.11, *p* = 0.79) (Fig. 4b).

**Table 7:** Statistical differences in the Th1/Th2 ratio with significant differences of control^−^ and SIDS cases.

Th1/Th2 ratios	Control^−^ (*n* = 10)		SIDS (*n* = 29)		Trends for SIDS	*p* value
	Median	Range	Median	Range		
IL-1β/IL-4	6.63	16.58	3.28	27.27	↓	**0.012**
IL-1β/IL-5	0.05	0.26	0.02	0.27	↓	**0.013**
IL-1β/IL-10	3.12	9.03	1.67	37.56	↓	**0.016**
IL-2/IL-4	2.58	14.86	1.57	2.69	↓	**0.021**
IL-2/IL-10	1.04	3.77	0.71	4.27	↓	**0.031**
CXCL8/IL-4	314.74	662.96	72.18	1002.30	↓	**0.013**
CXCL8/IL-5	1.75	10.50	0.54	12.57	↓	**0.005**
CXCL8/IL-10	132.69	367.72	33.91	1744.59	↓	**0.006**
TNF-α/IL-10	11.52	180.56	15.20	38.51	↑	**0.014**
TNF-α/IL-13	42.08	362.75	81.89	1250.08	↑	**0.013**

Note: A *p* value < 0.05 (two-tailed) was accepted as indicating statistical significance.

The Mann–Whitney *U*-test was used to compare two groups of continuous variables.

*Control*^*−*^ controls without signs of infection, *SIDS* sudden infant death syndrome.

## Discussion

Slight infection in the respiratory tract is frequently observed in SIDS and there is speculation about an impaired inflammatory response,^7^ but this potential relationship is still debatable and not fully understood.^4,7,25^ We performed a multiplex analysis of SIM levels in the lung tissue from SIDS and control cases to gain insight into the possible role of the pulmonary inflammatory response. We found decreased cytokine concentrations (IL-1RA, IL-7, IL-13, G-CSF) and a shift towards a Th2-dominated pattern in SIDS. For the first time, we demonstrate the lack of an age-dependent increase of the mean SIM production in SIDS lungs as opposed to control lungs (Fig. 4a) and an age-dependent shift towards Th2-related response in SIDS but not in controls (Fig. 4b).

Initially, we observed no differences between SIDS cases with mild upper airway infections (SIDS^+^) and those without any signs of infection (SIDS^−^) except for the elevation of CCL5 in the SIDS^+^ cases. CCL5 is a key pro-inflammatory chemokine, typically activated late (3–5 days) after T cell activation. Up-regulated levels of CCL5 are observed in most respiratory viral infections.^26^ The increased concentration of CCL5 is the only parameter in concordance with the upper airway infections reported for the SIDS^+^ group. Nevertheless, as other pro-inflammatory mediators (e.g., IL-2, IL-6, IFN-γ, CXCL10, CXCL8) that are involved in CCL5- mediated effects were not increased, the consequence of this finding is unclear and needs to be further investigated in larger cohorts. For further analysis, since SIDS^−^ and SIDS^+^ samples showed no overall statistical significance, both groups were merged into one (“SIDS”).

When comparing the Control^−^, Control^+^, and pooled SIDS groups, almost all cytokines, except CCL5, were massively increased in the Control^+^ group, supporting the concept that lungs of infants are able to mount robust inflammatory responses to infection. Therefore, this Control^+^ group confirms the reliability of the protein-based method of our study. Nevertheless, for further analysis only Control^−^ and SIDS groups were compared.

Prior studies on cytokine levels^27–31^ are scarce and contradictory. Vege et al. reported increased IL-6 in the cerebrospinal fluid (CSF) and elevated IL-6 receptor (IL-6R) in the arcuate nucleus,^28–30^ but no significant differences of IL-1β, IL-6, and TNF-α in CSF and serum were demonstrated by another group.^27^ However, decreased immune-related gene expression was revealed using a whole-genome gene expression assay by Ferrante et al.^31^ In this study, a down-regulated expression of myeloid differentiation primary response 88 (MyD88), an important signaling component of toll-like-receptors (TLR) in the brain, as well as CCL3 and Protein Unc-13 Homolog D (UNC13D) in the liver, were observed, implying a peculiar form of immunodeficiency. Lung tissue was not included in this study. Although we did not test UNC13D or MyD88 and found no differences between SIDS and control^−^ for CCL3 concentrations, the lack of responses to infection in our SIDS lung samples would support the idea of insufficient pathogen responses.^31^

For SIDS lungs, we describe herein, for the first time, a down-regulation of inflammatory SIMs, as four inflammatory mediators (IL-1RA, IL-7, IL-13, and G-CSF) were significantly decreased (Table 6). Even more so, we found a Th2-dominated shift of the cytokine milieu in the lungs that might contribute to pulmonary failure in SIDS. This finding strongly supports the hypothesis that a peculiar modification of the immune response to pathogens as well as in the immune homeostasis is implicated in the etiology of SIDS.

These findings are in accordance with an abstract reporting that inflammatory cells expressing Th2 cytokines might be increased in lung tissue of SIDS.^13^ Unfortunately, the authors apparently have never published these data as full article and presented neither original data nor a detailed discussion. Nevertheless, from our findings of a Th2-shifted cytokine milieu, the question arises whether or via which mechanisms this phenomenon might increase the risk of an infant to succumb to SIDS. In another context, such a shift is extensively observed in neonates who are susceptible to pathogens and at risk to develop allergic reactions.^32^ One approach to explaining the etiology of SIDS is the allergy to bacterial toxins: It is speculated that non-antibody-regulated induction of mast cell degranulation, leading to anaphylaxis, is activated in SIDS via increased IFN-γ induced by *pyrogenic staphylococcal* toxins.^7^ However, we did not find elevated IFN-γ concentrations in SIDS lungs. Furthermore, anaphylaxis triggered by degranulation of mast cells is usually accompanied with elevated IL-4 and IL-13^33^ (which were not elevated in our study) rendering an allergic reaction rather unlikely in our cases. Therefore, up to now, it is still unclear how a Th2 shift and the connected dysfunctional immune response might contribute to SIDS and further studies are needed to explore these interconnections.

Another interesting finding in our study appears to be the correlation between age and both pulmonary cytokine levels and Th1/Th2 balance. During infancy, the immune system is constantly maturing and both retardation and acceleration of this process might be deleterious.^34^ This maturation process linked to a “trained” immunity increasingly in contact with and adapting to external influences leads to an increase in cytokine production capacity during the first year of healthy infants.^35,36^

Nevertheless, up to now, no studies investigating the relationship between inflammatory mediators and age have been performed in SIDS. We observed that the concentrations of ten SIMs in SIDS or (and) controls were positively linked with increasing age (Fig. 3). However, this increase is less steep in SIDS than in controls. This observation can also be demonstrated for the normalized average mean-centered scaled cytokine concentrations (Fig. 4a): The overall cytokine concentrations were positively correlated with increasing age in the Control^−^ group, but not in the SIDS group. The results in the Control^−^ group were thus generally in line with the expected maturation process, but the SIDS group showed a retarded maturation process. As stringently stated in the triple risk hypothesis,^2^ there are vulnerable phases in the development of (some) infants during which stress that otherwise is of little consequence, might lead to SIDS. In line with our results, we thus propose that a part of SIDS cases seems to be related to an impaired maturation of the immune system that might have an influence on this vulnerable phase.

Interestingly, also the Th1/Th2 balance (mean-centered scaled ratios) was shifted to Th2 activation with increasing age in SIDS, but not in the Control^−^ group (Fig. 4b). This age-dependent shift might be interpreted as consequence of an up to now unknown impaired development of the extremely complex immune system in SIDS, e.g., by a hitherto not characterized immune deficiency. However, there is also another explanation by the evidence that children that later succumb from SIDS are subjected to chronic stress.^37,38^ The reasons for this stress might be manifold: E.g., sleeping in a prone position or an impairment of the serotonergic system.^37^ It is well known that chronic stress can lead to a shift from Th1 to Th2 immune responses.^39^ We, thus, tend to propose that the increase in a Th2-guided response observed in our cases may be, at least in part, a consequence of chronic stress for the exposed infants, although the possibility remains that it reflects a hitherto not elucidated primary problem of the immune system. Whatever the reason for this Th2-guided shift may be, this aberration should mostly concern older infants (e.g., from 6 months on). However, most SIDS cases occur between the second and fourth month of life. But SIDS is considered a multi-etiology syndrome and we argue that our findings might be at least for a subgroup of SIDS (i.e., the older ones) of importance.

There were several limitations in this study. First, a limited number of controls could be included into this study. Second, only one sample from the lungs was investigated and, therefore, focal alterations could not be investigated in detail. In many cases inflammation in the lungs is localized rather than generalized. Despite a thorough and detailed histological examination, a single sample, although representative to a certain extent, is still subject to possible sampling error. Third, it is well known that infections can vary depending on the type of pathogen. Moreover, they are dynamic processes with mediator concentrations varying over the course of the infection. In the present study, SIDS cases with diverse kinds of mild infections and a wide range of disease duration are included, which may influence the assessment of the results. Fourth, although no statistical differences in postmortem intervals and sample storage periods were observed between four groups, our samples varied widely in these respects, which also might influence the levels of inflammatory mediators.

## Conclusion

In summary, our results revealed four decreased mediators in SIDS and a Th2-guided shift in SIDS. More importantly, for the first time, it was demonstrated that there seems to be no correlation between age and the concentrations in cytokines in SIDS as opposed to the Controls and that there may be an age-dependent Th2 shift in SIDS, but not controls. We conclude that an impaired maturation of the immune system, an insufficient response to respiratory pathogens and an immune response modulated by Th1/Th2 imbalance might play a possible role in triggering SIDS. These findings might be explained in part by chronic stress. Further studies focusing on the immune status should be carried out in the lung as well as in other organs or tissues as well as the circulating compartment of the blood.

## Supplementary information

Supplementary Table S1

## Data Availability

All data generated or analyzed during this study are included in the Supplementary Information file of the published article. In addition, all data are available from the corresponding author upon reasonable request.
